# A novel gallium oxide nanoparticles-based sensor for the simultaneous electrochemical detection of Pb^2+^, Cd^2+^ and Hg^2+^ ions in real water samples

**DOI:** 10.1038/s41598-022-24558-y

**Published:** 2022-11-23

**Authors:** Gehad Abd El-Fatah, Hend S. Magar, Rabeay Y. A. Hassan, Rehab Mahmoud, Ahmed A. Farghali, Mohamed E. M. Hassouna

**Affiliations:** 1 Chemistry Department, Faculty of Science, Beni-Suef University, Beni-Suef, 62514 Egypt; 2 Applied Organic Chemistry Department, National Research Centre (NRC), Dokki, Giza, 12622 Egypt; 3 Nanoscience Program, University of Science and Technology (UST), Zewail City of Science and Technology, Giza, 12578 Egypt; 4 Materials Science and Nanotechnology Department, Faculty of Postgraduate Studies for Advanced Sciences, Beni-Suef University, Beni-Suef, 62511 Egypt

**Keywords:** Environmental sciences, Chemistry

## Abstract

Differential pulse voltammetry (DPV) using gallium oxide nanoparticles/carbon paste electrode (Ga_2_O_3_/CPE) was utilized for the simultaneous detection of Pb^2+^, Cd^2+^ and Hg^2+^ ions. Ga_2_O_3_NPs were chemically synthesized and fully characterized by Fourier-transform infrared (FTIR), X-ray diffraction (XRD), transmission electron microscopy (TEM) and scanning electron microscopy (SEM). Through the assay optimization, electrochemical screening of different nanomaterials was carried out using the cyclic voltammetry (CV) and electrochemical impedance spectroscopy (EIS) in order to determine the best electrode modifier that will be implemented for the present assay. Consequently, various parameters such as electrode matrix composition, electrolyte, deposition potential, and deposition time were optimized and discussed. Accordingly, the newly developed sensing platform showed a wide dynamic linear range of 0.3–80 µM with detection limits (LODs) of 84, 88 and 130 nM for Pb^2+^, Cd^2+^ and Hg^2+^ ions, respectively. While the corresponding limit of quantification (LOQ) values were 280, 320 and 450 nM. Sensors selectivity was investigated towards different non-targeting metal ions, whereas no obvious cross-reactivity was obtained. Eventually, applications on real samples were performed, while excellent recoveries for the multiple metal ions were successfully achieved.

## Introduction

In response to the severe warning from global warming and climate changes, environmental pollution caused by heavy metals has attracted large attention especially in recent decades. Among the list of heavy metals, Pb^2+^, Cd^2+^ and Hg^2+^ ions have a very hazardous impact on the environment, especially human health^1^. Those toxic ions have accumulative action on human organs; once they are absorbed, they are accumulated in body organs causing serious effects^2^. Exposure to Pb^2+^ ions causes many serious effects as it prevents hemoglobin formation, indigestion, anemia, high blood pressure, damages the immune system and kidneys; also causes harmful effects on children’s bones^3,4^. Cadmium (II) is considered as one of the most toxic heavy metals. Absorption of Cd^2+^ ions in the human body causes swelling of kidneys, liver and lungs^5–7^. Moreover, cadmium ions have severe effects on cardiovascular, immune and reproductive systems^8^. Mercury(II) is one of the oldest known contaminants; exposure to such toxic heavy metal, even at low concentrations, causes dangerous problems to the central nervous system, cause skin rashes and damage vital human organs^9^.

According to the updated standard regulations and guidelines of the World Health Organization and US Environmental Protection Agency guidelines, maximum allowable concentration of such heavy metal ions in drinking water should not exceed 1.0 µg/L^10^.

Thus, various analytical techniques have been developed for the determination traces amounts of heavy metal ions including the flame atomic absorption spectrophotometry (FAAS)^11^, electrothermal atomic absorption spectrometry (ETAAS)^12^, inductively coupled plasma mass spectrometry (ICP-MS)^13^, atomic fluorescence spectrometry (AFS)^14,15^ and high performance liquid chromatography (HPLC)^16^. Although these advanced techniques provide low detection limits and have the ability to measure many of metal ions simultaneously, they are very expensive, need sophisticated instruments, high sample consumption, need treatments for sample analysis, difficult procedures and long time for analysis. Hence, various electrochemical methods were developed to overcome the limitations of previous techniques. Electrochemical methods are preferred for providing accurate measurements, short time for the species detection, easy procedure, and cheap instrumentation, portable and user-friendly^17–22^. DPV technique is one of the electrochemical techniques which used for quantification of species with high accuracy, sensitivity and low detection limit. Electrode surface modification with nanomaterials (metal or metal oxides nanostructures) displayed a great impact on the selectivity and sensitivity of the electrochemical methods^23^.

Metal oxides (MOs) are prepared with different morphologies and crystalline structures, thus involving a large variety of potential applications^24,25^. In particular, MOs exist in the form of p and n-type semiconductors, which contain many oxygen vacancies^26^. These oxygen vacancies co-existed in MOs during the electrochemical applications are acting as charge carriers^27^. Predominant use for MOs, especially transition metals in electrochemical detection, is attributed to their electrocatalytic properties.

Gallium oxide (Ga_2_O_3_) is emerging as an essential material for optical devices, gas sensors and power electronic devices, due to the optoelectronic transparency, the large band gap, and the high breakdown voltage, combined with high electrical conductivity^28,29^. Ga_2_O_3_ nanoparticles (NPs) have a band gap of 4.8 ~ 5 eV, also have chemical and thermal stability that makes them a good participant in sensing applications^.^ Besides, the highly electrochemical performance of Ga_2_O_3_ NPs is attributed to its high electrical conductivity, high ratio and large surface area that results from the morphology of nanostructures^30^. Thus, gallium oxide was tested and investigated as a material for chemical sensors and catalyzers, for phosphors and electroluminescent devices, for high-voltage and power electronics, and of course for transparent conductive coatings^31^. Regarding the biocompatibility and cytotoxicity, gallium oxide and gallium-doped material showed excellent material-bone integration with no sign of local toxicity or implant rejection. Systemic biocompatibility investigation did not indicate any sign of toxicity, with no presence of fibrosis or cellular infiltrate in the histological microstructure of the liver and kidneys after 56 days of observation^32^.

In the present study, Ga_2_O_3_NPs were chemically synthesized and subsequently fully characterized by FTIR, XRD, TEM and SEM/EDX. The electrochemical characterizations were performed using cyclic voltammetry (CV) and electrochemical impedance spectroscopy (EIS) whereas modified carbon electrodes were exploited as the working electrodes. Thus, this is the first time to use gallium oxide as a novel nanomaterial for constructing simple and highly sensitive simultaneous quantification of Pb^2+^, Cd^2+^ and Hg^2+^ ions. For Ga_2_O_3_-based sensor parameters optimization; electrode composition, types and concentration of the supporting electrolyte, deposition potential and deposition time were studied. The modified sensor was used for determination of Pb^2+^, Cd^2+^ and Hg^2+^ ions in real water samples.

## Methods

### Chemicals and reagents

For Ga_2_O_3_ nanostructures preparation, gallium metal was purchased from Fluka, aqueous diethylene glycol (DEG) and ammonium hydroxide (NH_4_OH) were purchased from Sigma-Aldrich. Besides Ga_2_O_3_ NPs, the synthesized MOs were also used in this current study including the tungsten dioxide (WO_2_), zirconium dioxide (ZrO_2_), nickel dioxide (NiO_2_) and cerium dioxide (CeO_2_). Different supporting electrolytes were prepared in double distilled water using acetic acid (HAc, 99.79%), nitric acid (HNO_3_, 68%), phosphoric acid (H_3_PO_4_, 85%), sodium chloride (NaCl) and potassium chloride (KCl). Acetate buffer solution of 0.1 M for pH 5.0 was prepared by mixing stock solution of 0.1 M sodium acetate (NaAc) and (HAc). Potassium ferricyanide and potassium ferrocyanide were purchased from Piochem for laboratory chemicals and ALPHA CHEMIKA, respectively. Cadmium nitrate (Cd(NO_3_)_2_), lead nitrate (Pb(NO_3_)_2_) and mercury chloride (HgCl_2_) were purchased from (Merck, Germany), (WINLAB, LABORATORY CHEMICALS REAGENTS FINE CHEMICALS) and ACROS ORGANICS, respectively. For carbon paste electrode preparation, graphite powder was provided by ACROS ORGANICS and paraffin oil was purchased from Fluka. In addition, interferences study was carried out using copper (II) nitrate (Cu(NO_3_)_2_.3H_2_O), zinc chloride (ZnCl_2_) and potassium chromate (K_2_CrO_4_) were purchased from (WINLAB, LABORATORY CHEMICALS REAGENTS FINE CHEMICALS), calcium carbonate (CaCO_3_), magnesium nitrate hexahydrate (Mg(NO_3_)_2_.6H_2_O), ferric nitrate (Fe(NO_3_)_3_.9H_2_O and chromium (III) nitrate (Cr(NO_3_)_3_.9H_2_O), were purchased from Piochem for laboratory chemicals, Sisco Research Laboratories, ALPHA CHEMIKA and Riedel–deHaen, respectively.

### Synthesis of gallium oxide nanomaterials

According to the previously published methods^33,34^, X gallium oxide nanoparticles were chemically synthesized as follows: di-ethylene glycol (DEG) was added drop by drop after the dissolution of gallium metal with strong stirring. The obtained solution was reacted with NH_4_OH solution with continuous stirring forming a precipitate of gallium hydroxide, which was treated hydrothermally in an autoclave at 180 °C for 24 h. The resulting product was washed with double distilled water, dried in an oven, followed by thermal treatment to 650 °C and finally kept at 650 °C for 5 h.

### Instruments for material characterizations

Electrochemical characterizations were conducted using a Potentiostat/Galvanostat (AUTOLAB-PGSTAT 302 N, Metrohm). The electrochemical cell was connected to three-electrodes whereas the modified carbon paste electrode was the working electrode, platinum wire (Pt) and Ag/AgCl were the counter and the reference electrode. The FTIR spectroscopic spectrum of Ga_2_O_3_NPs was recorded using Bruker Vertex 70. The XRD analysis was performed in the 2-theta range of 10 to 80° (Shimadzu, Kyoto, Japan), and verified via (Cu, wavelength 1.5406) AXS D8 Advance diffractometer). Size, morphology, and elemental composition of the metal nanostructures were determined and characterized using TEM (JEOL JEM-1230) and SEM/EDX (Gemini Zeiss-Sigma 500 VP).

### Fabrication of the modified sensor (Ga_2_O_3_/CPE)

Ga_2_O_3_-based electrodes were fabricated by homogenous mixing of Ga_2_O_3_NPs, graphite powder and 250 µL paraffin oil in a mortar for 30 min^35^. The resulting paste was carefully packed and compressed into a Teflon tube as a holder with a diameter of 5 mm, with a Cu wire as an electrical connector. Before using the electrode, its surface was polished on a smooth paper, finally rinsed with double distilled water.

### Electrochemical measurements

Cyclic voltammetry (CV) measurements were conducted in 0.1 M KCl electrolyte containing 5 mM of the K_3_[Fe(CN)_6_]^3/4^ at potential range of − 0.4 to 0.6 V with scan rate of 50 mV/s. Electrochemical impedance spectroscopy (EIS) measurements were performed at frequency range of 10 kHz to 0.1 Hz under 10 mV AC-potential amplitude at open circuit potential in KCl (0.1 M) containing 5 mM of the K_3_[Fe(CN)_6_]^3/4^. EIS analysis was carried out using the modeled equivalent circuit. Differential Pulse Voltammetry (DPV) measurements were performed in 0.1 M HNO_3_ as the optimum electrolyte with potential range of − 1.1 to 0.4 V. All obtained electrochemical responses were recorded at room temperature (25 ± 2 °C).

### Real sample analysis

Treated waste water sample has been kindly provided by Abu Shahba Sanitation (Beni-Suef Water & Sanitation Company, Public management of central laboratory WWTP). The sample has been checked by ICP (Perkin Elmer aveo220 max) before and after spiking with standard concentrations of the targeting metal ions. Tap water samples were collected from the local chemistry labs, and their heavy metal contents were also DPV analyzed after spiking with the metal ions. All recovery rates were calculated and presented accordingly.

### Statistics and data analysis

All data are presented as mean ± SD after considering at least three individual electrochemical testes. Statistical significance was estimated by statistical hypothesis testing, the significance of the values was assumed as *p* < 0.05. Each limit of detection (LOD) and limit of quantification (LOQ) values were calculated from calibration curves. The reproducibility and repeatability of the modified sensor were represented using the relative standard deviation (RSD). All of the represented plots were sketched by Origin Lab software.

## Results and discussion

### Characterization of the Ga_2_O_3_ NPs

**Figure 1:**
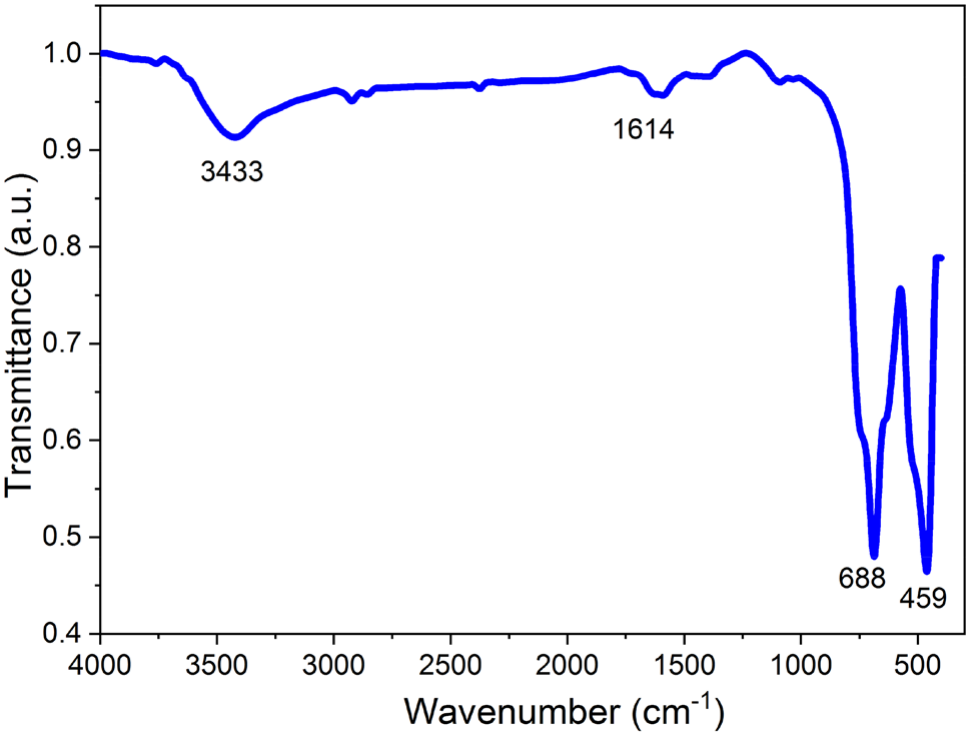
FTIR spectrum of the prepared Ga_2_O_3_NPs.

For the functional analysis, the FTIR spectrum of Ga_2_O_3_NPs was analyzed in the range of 400–4000 cm^−1^ as shown in Fig. 1. A broad absorption band was observed in the range of 3000–3700 cm^−1^. The band appearing at 3433 cm^−1^ was corresponding to H–O–H stretching. The weak peak at 1614 cm^−1^ represents the bending vibrations of –OH groups and adsorbed water molecules. Strong peaks at 688 cm^−1^ and 459 cm^−1^ as result of Ga–O bending vibrations^31^. Thus, from FTIR analysis, Ga_2_O_3_NPs are formed only in monoclinic structure.

**Figure 2:**
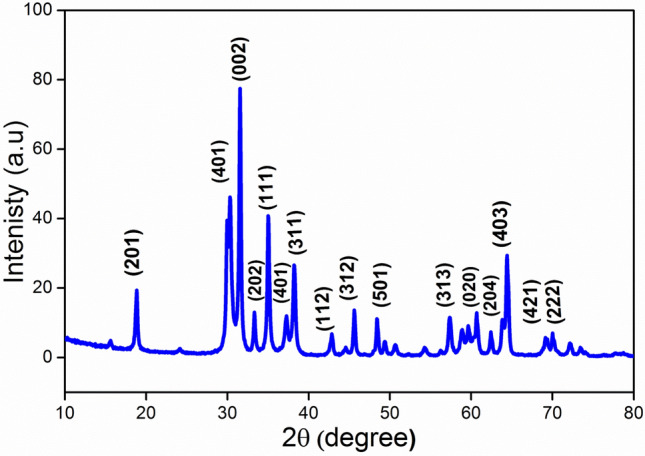
XRD of Ga_2_O_3_NPs.

**Figure 3:**
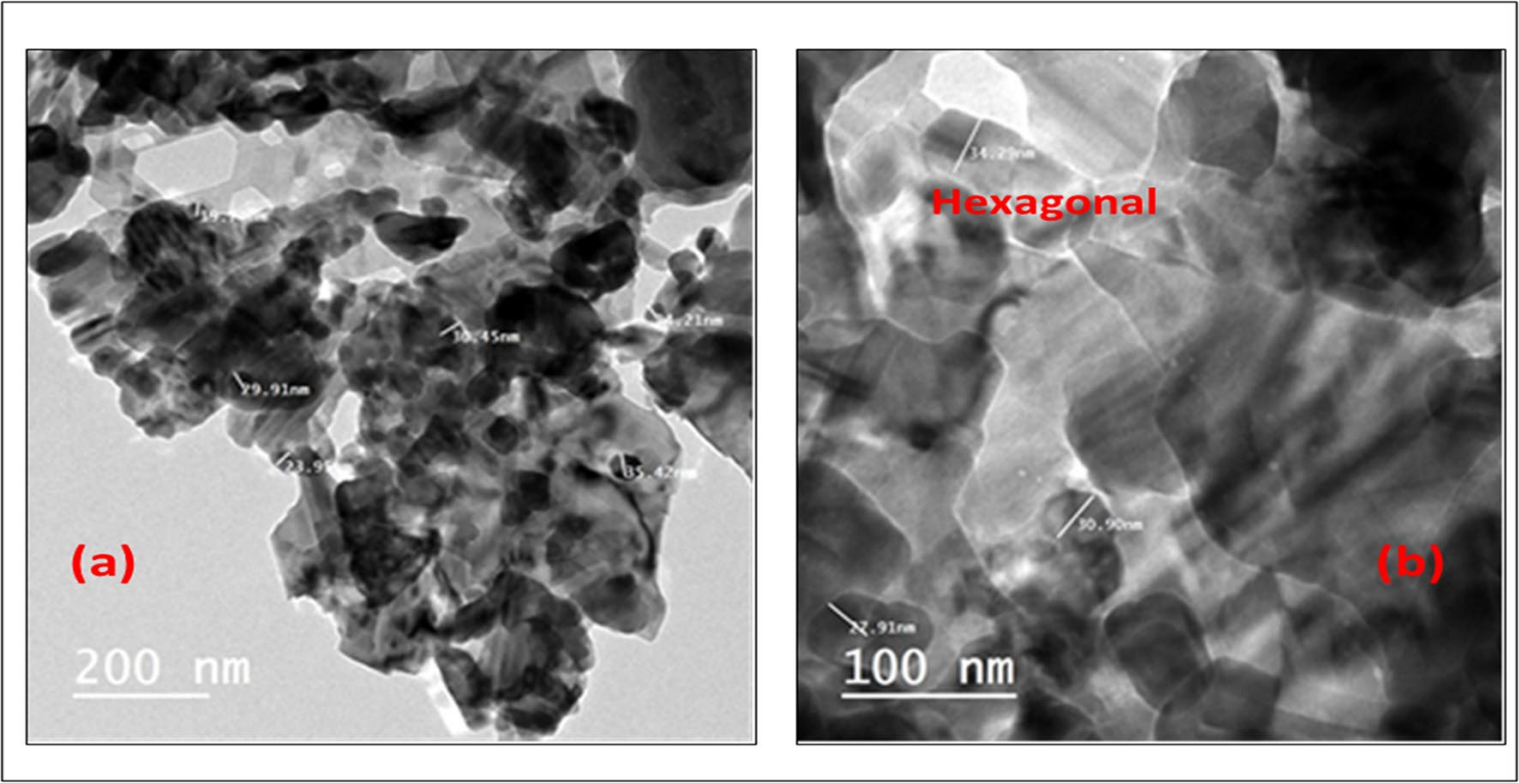
TEM images (**a**) and (**b**) of the synthesized Ga_2_O_3_NPs.

Furthermore, XRD analysis for the nanoparticles was carried out for crystalline, purity and atomic structure investigation, as shown in Fig. 2. Sharp and strong peaks were observed illustrating that gallium oxide diffraction powders are prepared with high crystalline structure, thus a characteristic diffraction peak with highest intensity was positioned at 2θ = 31.560 with d spacing = 0.283 nm. This clear diffraction peak corresponds to the following miller indices of plane (002) which indicate that pure gallium oxide crystals are obtained in the hexagonal phase. No impurity peaks were observed in the limit up to 80 degree, indicating the high purity of the prepared nanomaterials. The average crystallite size was estimated for the index of (002) using the formula of Debye–Scherrer^36^. The average size of Ga_2_O_3_ NPs was calculated to be 30.2 nm. From the XRD pattern, gallium-oxide NPs were formed in a single-phase structure. The morphological characterization of Ga_2_O_3_NPs using TEM analysis was utilized for particle sizes and shapes determination, where homogeneous hexagonal shapes and uniform nano-sizes ranged from19–34 nm as illustrated in Fig. 3 (a) and (b) which was demonstrated by the XRD as well.

**Figure 4:**
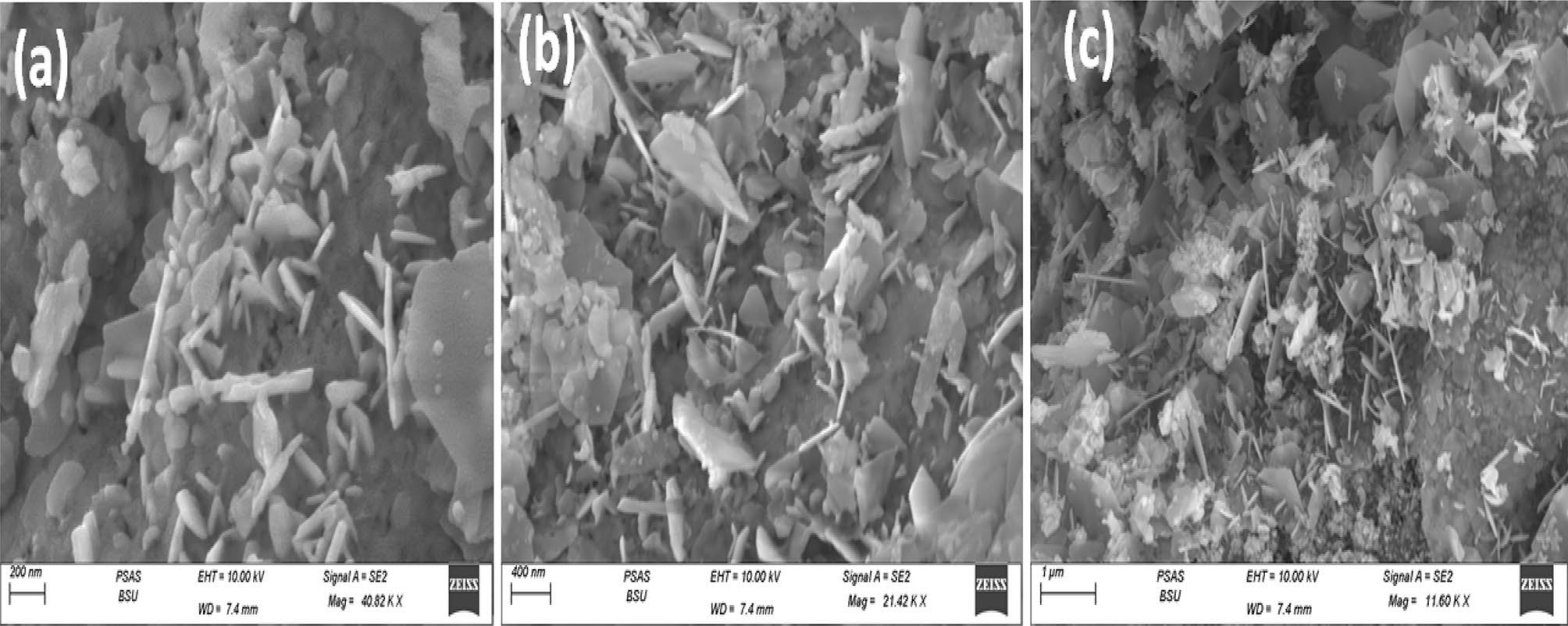
SEM images (**a**), (**b**) and (**c**) of Ga_2_O_3_NPs.

**Figure 5:**
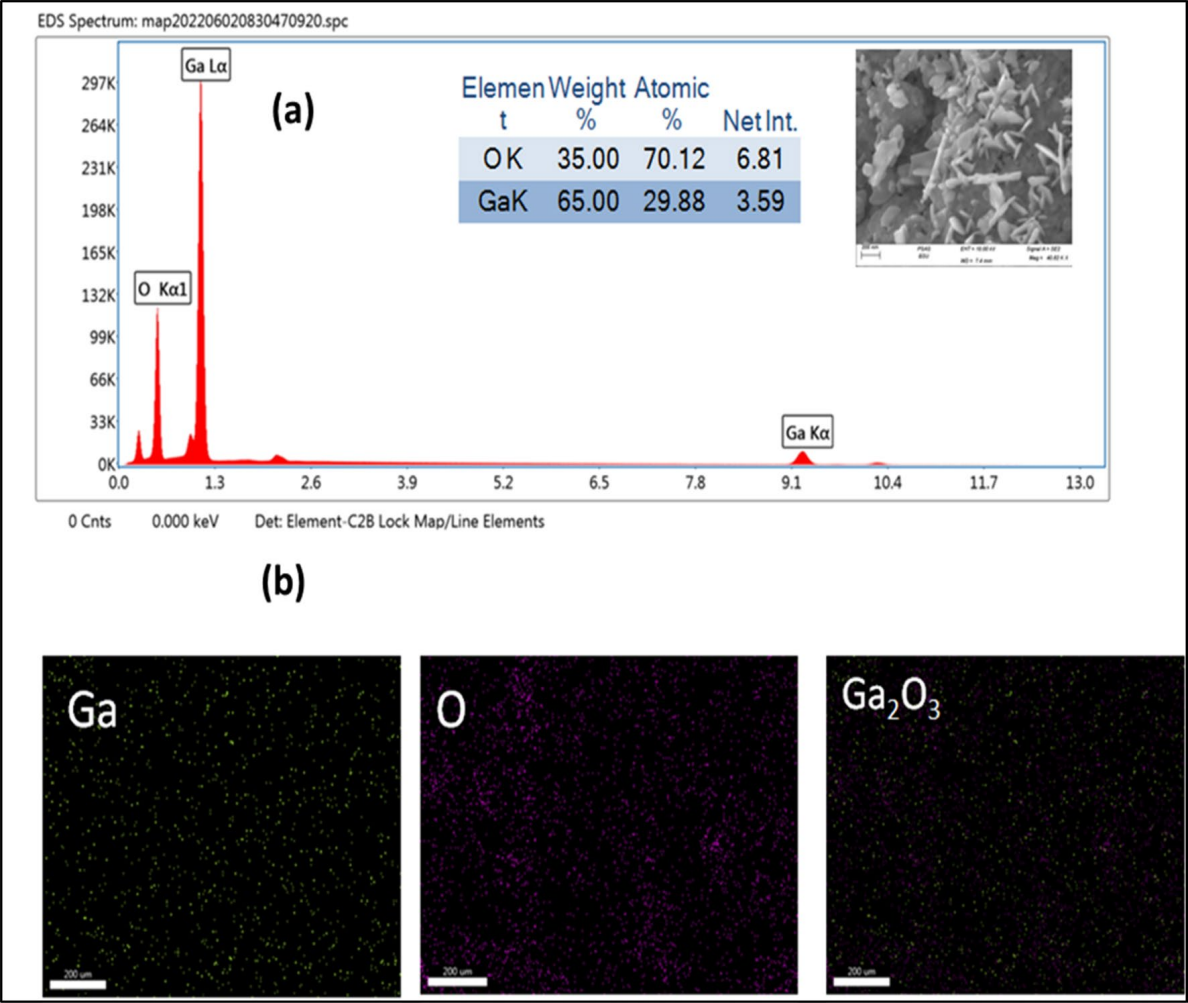
EDX (**a**) and elemental mapping (**b**) of Ga_2_O_3_NPs.

Morphology of Ga_2_O_3_ NPs was studied by the SEM analysis. Figure 4(a,b,c) displays the morphology of the synthesized Ga_2_O_3_NPs, where the particles are found as layers and spindle with average particle diameter from 22 to 30 nm as shown in Fig. 4(c). EDX and mapping analysis were used for the elemental and composition analysis as shown in Fig. 5(a,b). The percentage of elemental composition (Ga and O) was obtained by EDX and mapping analysis proved that gallium and oxygen are present in the percentages of 65% and 35%, respectively. Worth mentioning here that the pore structure of the Ga_2_O_3_ that was shown by the SEM images (Fig. 4(a)) could be exploited for the selective uptake of heavy metal ions, hence electrode surface modification with such nanomaterial will support the construction of a high performance electrochemical assay.

### Electrochemical investigation of different nanomaterials

**Figure 6:**
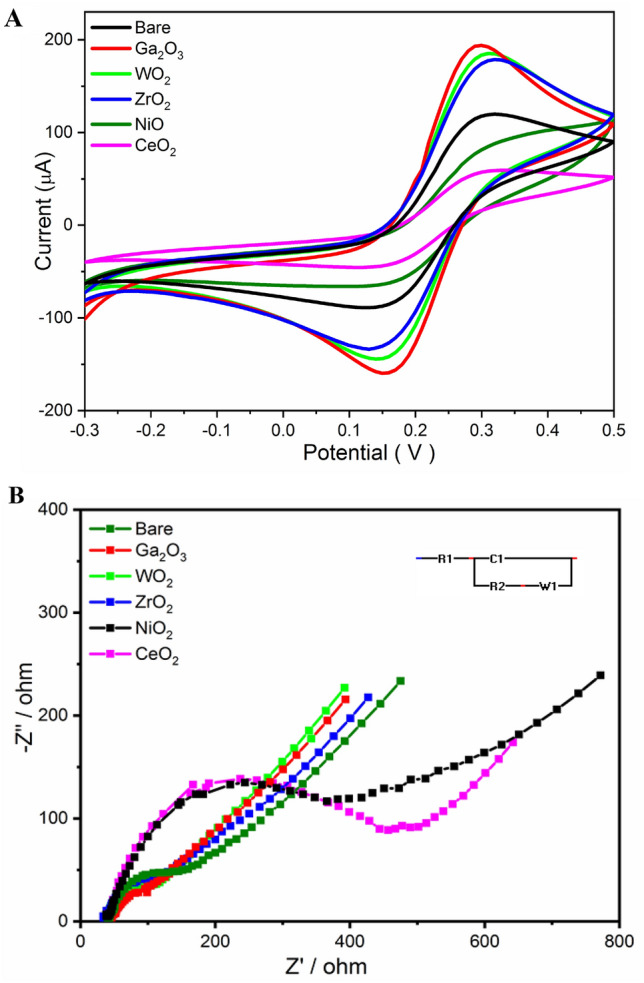
(**A**) CV and (**B**) EIS measurements for the unmodified and modified carbon paste electrodes with 30 mg of each metal oxide.

Here, modification of the electrode surface with different metal oxide nanostructures was suggested for providing a highly efficient electrochemical sensing platform. In this regard, several types of MOs including Ga_2_O_3_, WO_2_, ZrO_2_, NiO_2_ and CeO_2_ were screened. Accordingly, for each individual modified electrode, characteristics of both CV and EIS data were recorded in Ferricyanide (FCN) as the standard redox probe. As a result, electrochemical signals of the modified electrode with the Ga_2_O_3_ NPs demonstrated the highest redox peak heights than all other modified electrodes, as shown in Fig. 6(A). For the electrochemical impedance spectra, Nyquist plots shown in Fig. 6(B) confirmed the impact of the Ga_2_O_3 _nanomaterials on the enhancements of the electrochemical signal of their modified electrodes whereas the charge transfer resistance (R_ct_) was significantly dropped by the Ga_2_O_3_-modified electrodes. Table S1 (supplementary materials) showed the calculated and all obtained electrochemical information obtained by the CV and EIS analysis for all modified and unmodified electrodes. Thus, Ga_2_O_3_ NPs were selected and assigned for the voltammetric determination of multiple heavy metal ions.

### Voltammetric sensing of multiple heavy metal ions (Pb^2+^, Cd^2+^and Hg^2+^)

**Figure 7:**
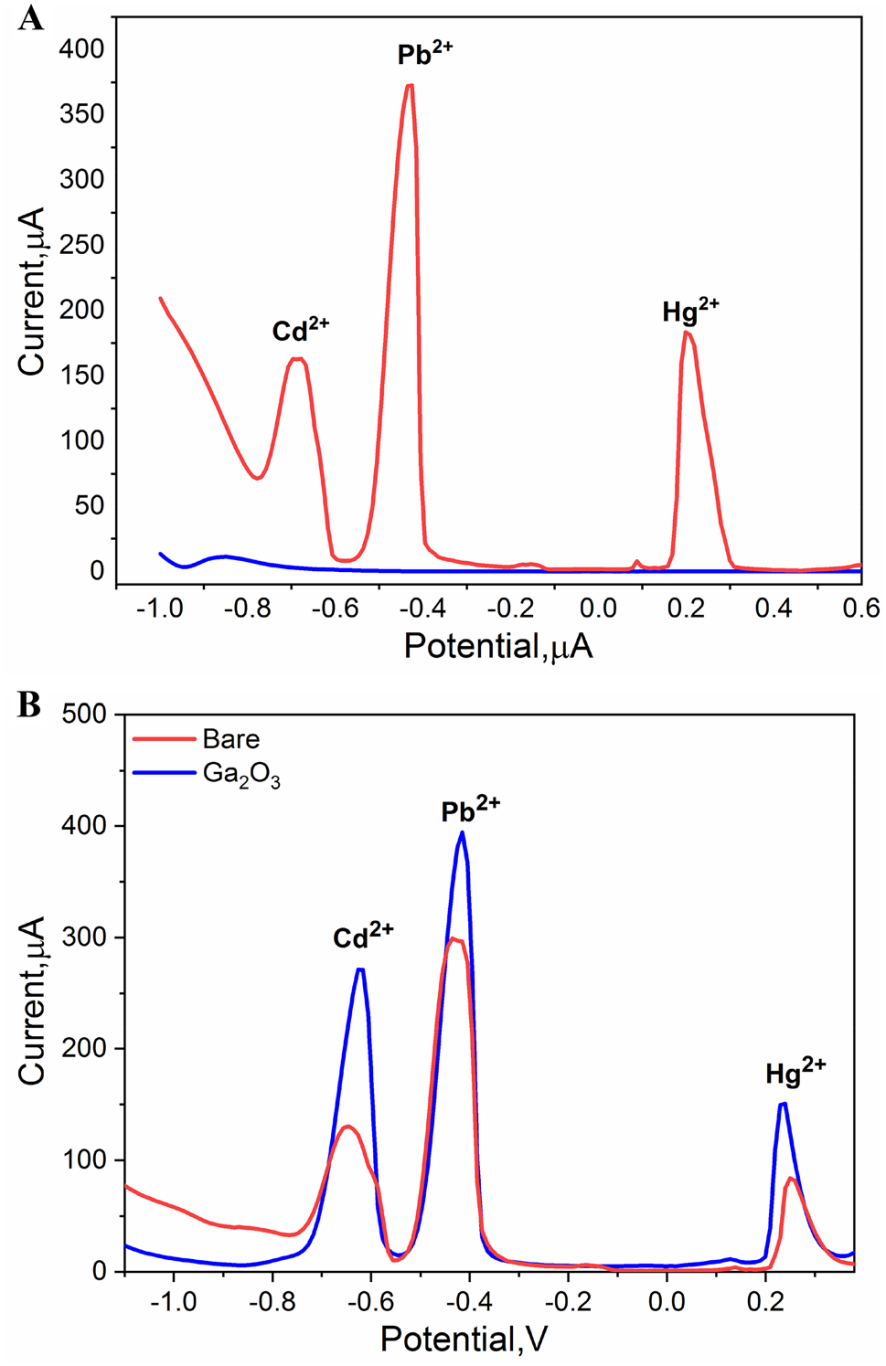
(**A**) DPV responses of Ga_2_O_3_-based electrode in the plain supporting electrolyte (blue line) which is the 0.1 M of the HNO_3_, and after injecting a mixture of the targeting metal ions (Pb^+2^, Cd^+2^ and Hg^+2^) with the concentration of 0.001 M of (red line). (**B**) DPV signals of Ga_2_O_3_/CPE compared with the bare electrode in 0.1 M HNO_3_ containing 0.001 M of each of Pb^+2^, Cd^+2^ and Hg^+2^ ions.

From the DPV measurements (Fig. 7A), anodic peaks of Pb^2+^, Cd^2+^ and Hg^2+^ ions appeared at designated potentials of − 0.42 V, − 0.63 V and + 0.24 V, respectively. In respect to the anodic peak heights, two distinguished responses towards the heavy metals were received from the unmodified or the Ga_2_O_3_ modified electrodes whereas an increase in several orders of magnitude was obtained by the modified surfaces, as depicted in Fig. 7B. This synergistic enhancement is attributed to the high electro-catalytic activity of Ga_2_O_3_NPs as well as the expanded electrode surface due to the addition of nanomaterial into its matrix.

As a proposed sensing mechanism, at the beginning of the electrochemical reaction, heavy metal ions in the cationic forms are adsorbed to the electrode surface via the electrostatic interaction with the negatively charged surface of the Ga_2_O_3_NPs as follows:

$${\text{M}}^{{{2} + }} + {\text{Ga}}_{{2}} {\text{O}}_{{3}} \to {\text{M}}^{{{2} + }} - {\text{Ga}}_{{2}} {\text{O}}_{{3}} \ldots \ldots_{{{\text{adsorption}}}}$$

Then, the adsorbed cationic metal ions will be electrochemically reduced to M^0^ under the applied voltammetric potential, which is then will be oxidized to generate the anodic peak currents.

### DPV assay optimization

For obtaining high sensitivity, several parameters and experimental conditions are optimized and in the next subsections, their details will be discussed.

### Effect of electrode composition

**Figure 8:**
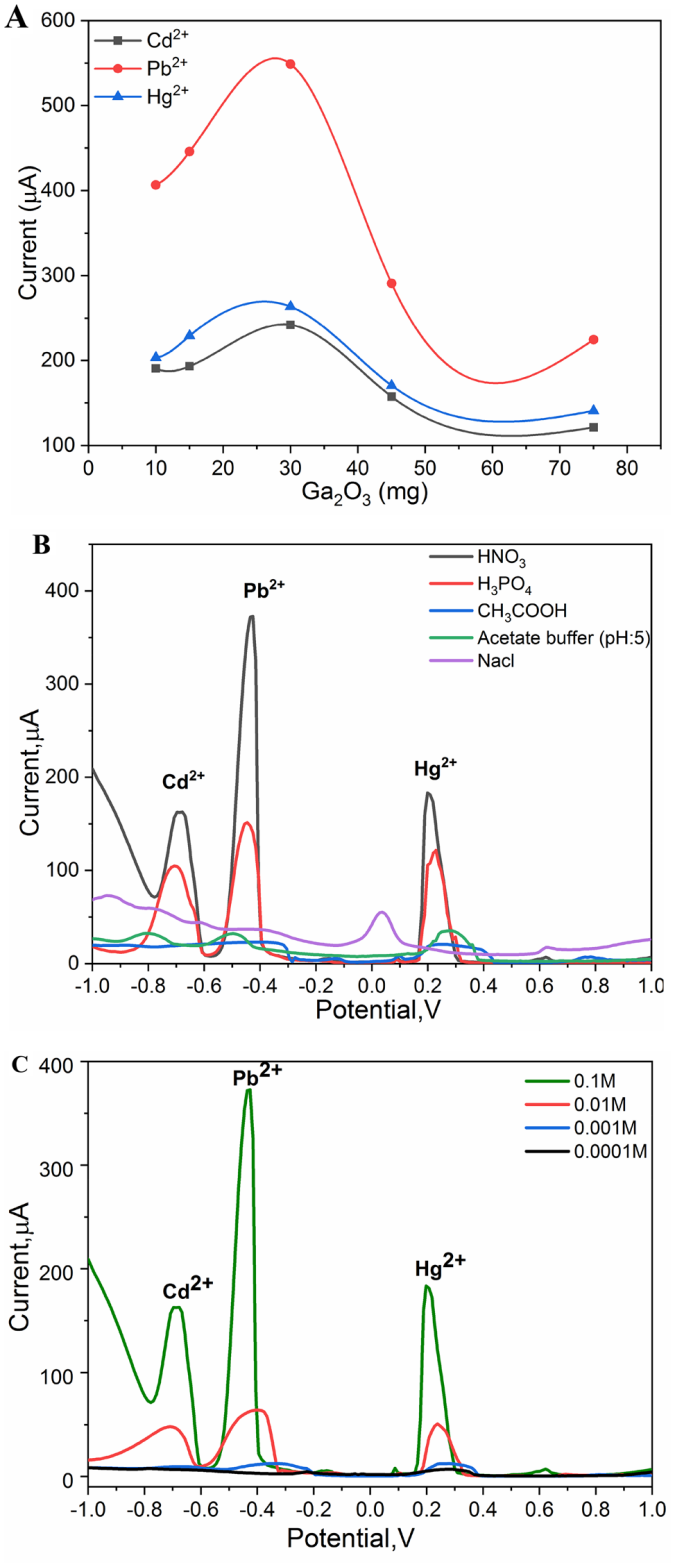
(**A**) DPV response of Ga_2_O_3_-based electrode with different concentrations in the electrode matrix. DPV measurements were conducted in 0.1 M HNO_3_ containing 0.001 M of each of Pb^+2^, Cd^+2^ and Hg^+2^ ions. (**B**) DPV measurements of Ga_2_O_3_-based electrodes in various supporting electrolytes (0.1 M), each electrolyte containing 0.001 M of each of Pb^+2^, Cd^+2^ and Hg^+2^ ions. (**C**) DPV response of Ga_2_O_3_-based electrode in different concentrations of HNO_3_ as the optimum supporting electrolyte, each concentration containing 0.001 M of each of Pb^+2^, Cd^+2^ and Hg^+2^ ions.

Usually nanomaterial content or concentration in any sensing and bio-sensing platform have a certain limit and capacity which should not be exceeded in order to satisfy the desired needs for high sensitivity and selectivity. Thus, impact of Ga_2_O_3_ NPs concentration into the electrode matric was studied at different concentrations ranging from 10 to 90 mg, while the graphite-powered content was constant in all modified electrodes. The performance of each of the modified electrodes was tested individually towards the voltammetric oxidation of the selected three metal ions, as shown in Fig. 8A. As a result, the influence of nanomaterial concentration was clear and the increase of the oxidation peak current was obtained for all metal ions. However, increasing the Ga_2_O_3_NPs concentration over 30 mg disturbed the DPV signals; hence, the oxidation current was significantly dropped. Since the highest signal was obtained when the Ga_2_O_3_NPs concentration was 30 mg, it  was applied for the next optimizing parameters.

### The influence of supporting electrolytes

For further optimization, DPV performance of the Ga_2_O_3_-based electrode towards the oxidation of the targeting metal ions was tested in different supporting electrolyte solutions including H_3_PO_4_, HNO_3_, CH_3_COOH, sodium chloride and acetate buffer. As shown in Fig. 8B, sharp and defined three anodic peaks were clearly generated when nitric and phosphoric acids were implemented as supported electrolytes. However, the highest electrochemical signals were supported by the nitric acids. The other tested electrolytes did not support the efficient oxidation of the targeting metal ions. Thus, nitric acid was selected. Consequently, different concentrations of nitric acid were examined, as shown in Fig. 8C. The performance of the Ga_2_O_3_-based electrode was strongly affected by the change of nitric acid concentration, whereas 0.1 M exhibited the highest DVP signals for all metal ions.

### Effect of deposition potential and accumulation time

Deposition potential is one of the crucial factors that influence the rate of the deposition of the targeting analyte(s) into the interface or the electrode surface. Therefore, it is worthwhile to test the DPV signals at different deposition potentials. In that regard, a potential range of − 1.3 to − 0.9 V was applied and the oxidation of the targeting metal ions was tested. Resulted data (Fig. 1S, Supplementary materials) showed that the increase in the peak currents of all metal ions (Pb^2+^, Cd^2+^ and Hg^2+^) was dependent on the increase of the accumulation potential until reaching a potential of − 1.1 V, then a drop in the oxidation current was obtained.

Consequently, the deposition time, which is needed for enriching the metal ions into the modified electrode surface, was studied from 30 to 120 s at − 1.1 V as the selected applied potential. As a result, the increase in the peak currents was correlated with the increase in the time of deposition until reaching 60 s (Fig. 2S, Supplementary materials). Therefore, accumulation potential and time of − 1.1 V, and 60 s were chosen as the optimum parameters.

### Standard calibration curves

**Figure 9:**
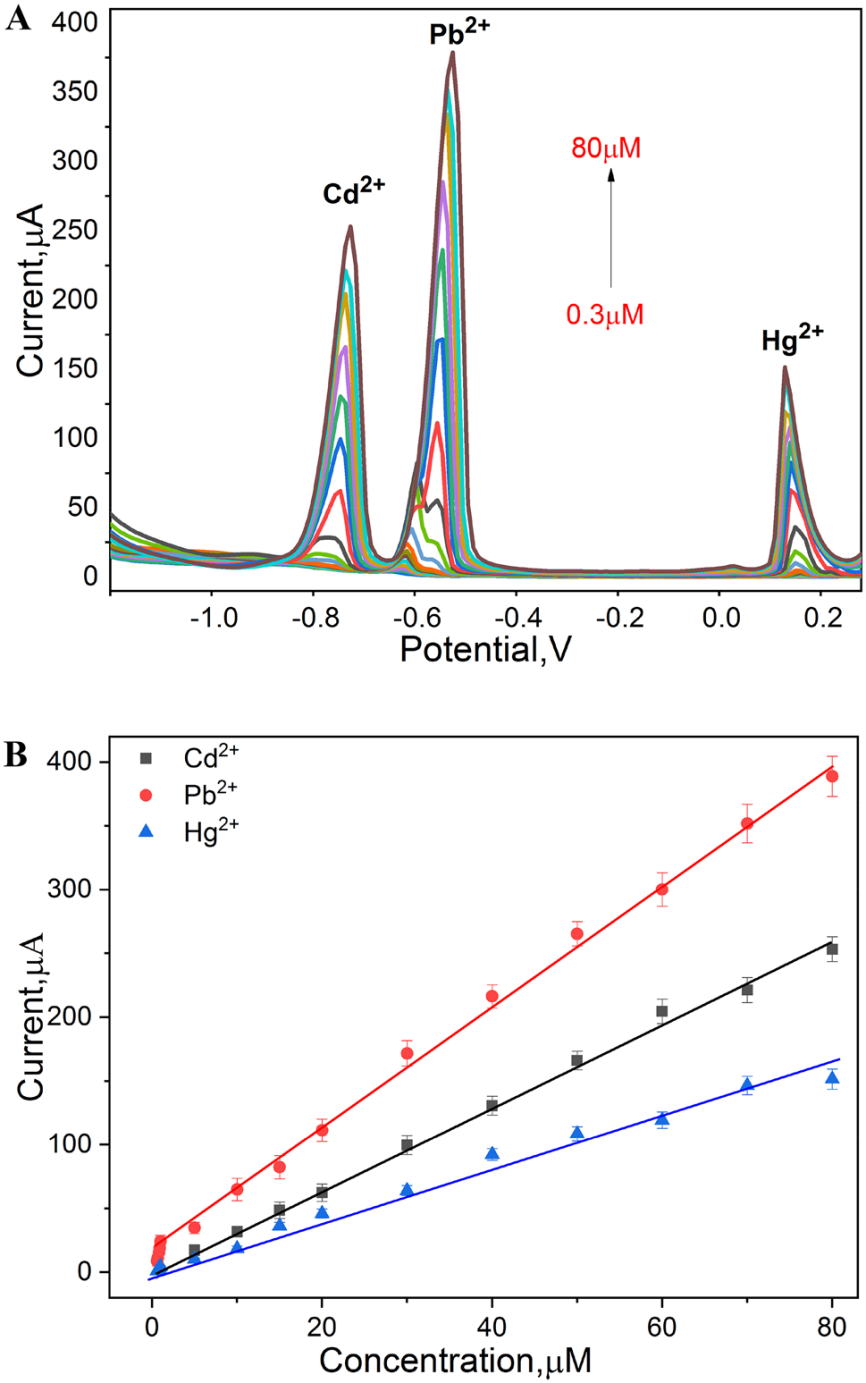
(**A**) DPV peaks of Ga_2_O_3_-based electrode of Pb^+2^, Cd^+2^ and Hg^+2^ ions over concentration range of 0.3–80 µM, at a fixed deposition potential of − 1.1 V, equilibrium time 15 s and step potential of 0.01 V. (**B**) Linear regression lines between concentration and corresponding oxidation current values of Pb^+2^, Cd^+2^ and Hg^+2^ ions.

After optimizing all of the experimental conditions, Ga_2_O_3_-based electrodes were exploited for the simultaneous quantitative analysis of Pb^2+^, Cd^2+^ and Hg^2+^ ions. For determining the capacity of linearity and sensitivity towards each metal ion, a continuous standard addition of mixture of Pb^2+^, Cd^2+^ and Hg^2+^ ions was conducted. As a result, very sharp and defined oxidation peaks with a gradual increase that was strongly dependent on the addition of the metal ions were obtained as depicted in Fig. 9A. The determined calibration curves for each metal ion covered the entire range of concentrations from 0.3 to 80 µM, 5 to 80 µM and from 0.6 to 80 µM for the Pb^2+^, Cd^2+^ and Hg^2+^, respectively (Fig. 9B). From the statistical analysis, correlation coefficients (R^2^) of 0.989, 0.99 and 0.972 were obtained with limit of detections of 84 nM, 88 nM and 130 nM, and limit of quantifications of 280 nM, 320 nM and 450 nM, respectively.

Referring to the previously reported data regarding the electrochemical detection of the selected metal ions, Table 1^37–41^ summarized the most updated limit of detections and the nano-materials used for the electrode modifications. As shown in that table, the highest sensitivity is exhibited by the newly developed Ga_2_O_3_-sensor.

**Table 1:** A list of previously published electrochemical methods for the simultaneous detection of heavy metal ions (Pb^2+^, Cd^2+^ and Hg^2+^). The present method is included for comparison.

Electrode	Technique	LOD, nM			Refs
		Pb^2+^	Cd^2+^	Hg^2+^	
EDTA_PANI/SWCNTs/SS	DPV	1650.0	–	680.0	^37^
BiNP/Nafion-modified PGE	ASV	149.95	65.03	–	^38^
CeHCF/GCE	LSV	9.65	88.95	14.95	^39^
PGMGPE	CV	800.0	–	6600.0	^40^
polyPCA/GE	SWASV	65.64	136.99	–	^41^
Ga_2_O_3_/CPE	DPV	84.0	88.0	130.0	Current method

### Interferences study

To investigate the selectivity of Ga_2_O_3_ sensor towards Pb^2+^, Cd^2+^ and Hg^2+^ ions, the Ga_2_O_3_-based electrode was tested in the presence of other metal ions including the Mg^2+^, Fe^3+^, Cr^6+^, Cr^3+^, Zn^2+^, Ca^2+^ and Cu^2+^. This cross-reactivity test was carried out individually at a single concentration (50 µM) of the ions. As a result, Table 2 demonstrated that the Mg^2+^ and Ca^2+^ have slight negative interference effect by ≈ 5%, while the Cr^6+^ and Zn^2+^ have affected the DPV signals of the targeting ions with about 30% and Cu^2+^ ions have a positive interference by ≈ 20%.

**Table 2:** Effect of the interfering ions on the differential pulse output signals of target ions.

Interferring ions	Concentration (µM)	Peak currents (µA)			Signal change %		
		Pb^2+^	Cd^2+^	Hg^2+^	Pb^2+^	Cd^2+^	Hg^2+^
Blank	50	282.22	100.86	70.9	–	–	–
Mg^2+^	50	268.86	68.9	51.36	− 4.73	− 31.7	− 27.6
	110	264.22	81.91	57.27	− 6.37	− 18.8	− 19.2
Fe^2+^	50	236.31	92.04	106.77	− 16.3	− 8.74	50.6
	110	217.91	88.91	96.45	− 22.8	− 11.8	36.03
Cr^6+^	50	189.31	86.81	98.5	− 32.9	− 13.9	38.9
	110	181.77	80.81	101.9	− 35.6	− 19.9	43.7
Cr^3+^	50	286.63	115.09	81.04	1.56	15.01	14.3
	110	285.22	124.81	95.18	1.06	24.4	35.2
Zn^2+^	50	185.77	47.95	120.36	− 34.2	− 52.5	69.8
	110	150.31	72.22	284.0	− 46.7	− 28.4	100.0
Ca^2+^	50	246.81	82.13	52.71	− 12.5	− 18.6	− 25.7
	110	319.04	115.9	71.86	− 13.0	14.9	1.35
Cu^2+^	50	340.9	82.40	44.22	20.8	− 18.3	− 37.6
	110	136.4	0	63.09	− 51.6	− 100.0	− 11.0

### Reproducibility and repeatability of Ga_2_O_3_ sensor

Four freshly prepared Ga_2_O_3_-based electrodes were prepared and the DPV response of each of them was individually tested towards a mixture of the target metal ions at 50 µM. Very close responses were received from all prepared electrodes with slight variations (1.99, 4.33 and 5.73% for Pb^2+^, Cd^2+^ and Hg^2+^, respectively). Thus, a high reproducibility was attained.

On the other hand, repeatability of Ga_2_O_3_-based electrodes was evaluated whereas DPV measurements were conducted separately five times using only one modified electrode. As a result, relative standard deviations (RSDs) of 4.77, 4.57 and 6.8, were obtained for Pb^2+^, Cd^2+^ and Hg^2+^ ions, respectively. Accordingly, a high repeatability was attained as well. Thus, the main criteria (i.e. the selectivity, sensitivity, reproducibility and repeatability) for designing a high effective chemical sensor for multiple and simultaneous analyte (s) detection was successfully achieved.

### Real sample analysis

**Figure 10:**
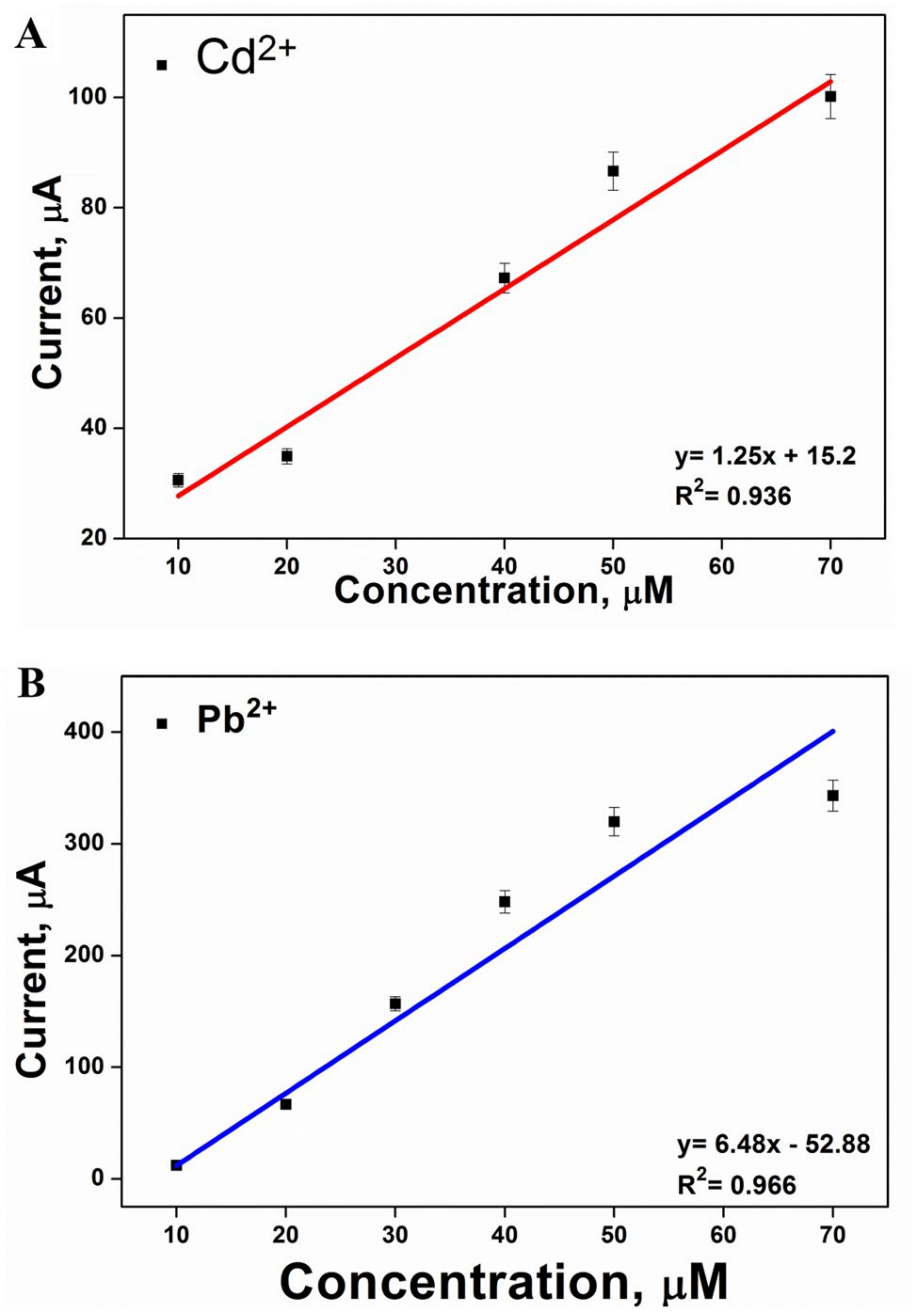
(**A**) and (**B**) represent the regression lines of Cd^+2^ and Pb^+2^, determined with spiked treated wastewater samples using the Ga_2_O_3_-based electrode.

Waste water sample containing traces of five metal ions (Cd^2+^, Cu^2+^, Pb^2+^, Ni^2+^ and Zn^2+^) as illustrated in Table 3 was considered for the direct DVP analysis using the newly modified chemical sensor. Since these concentrations are lower than the detection limits of the fabricated Ga_2_O_3_ sensor, it was an obligation to apply the spiking of concentrations from (10 to 70 µM) of Cd^2+^ and Pb^2+^ ions to the real treated sample to investigate the applicability of the electrode for the analysis of environmental samples. Table 3 showed that the recoveries of Cd^2+^ and Pb^2+^ ions using Ga_2_O_3_-based electrode were in the range of 95–110%. The regression lines obtained were displayed in Fig. 10A,B showed the excellent linear relationships between the concentration of each of the target ions and the corresponding anodic peak current values.

**Table 3:** Analysis of real treated wastewater samples, previously monitored by ICP, before and after spiking with Cd^2+^ and Pb^2+^ ions using Ga_2_O_3_-based sensor.

Technique	Real treated wastewater sample	Added (µM)	Found (µM)	Recovery %
ICP	Cd^2+^	–	0.48	–
	Cu^2+^	–	0.44	–
	Pb^2+^	–	0.02	–
	Ni^2+^	–	0.30	–
	Zn^2+^	–	0.62	–
DPV (Ga_2_O_3_/CPE)	Cd^2+^	10	9.65	96
	Cd^2+^	40	39.77	99
	Cd^2+^	70	66.78	95
	Pb^2+^	30	32.65	109
	Pb^2+^	70	76.94	110

Additionally, spiked (synthetically contaminated) tap water samples were analyzed, while the calculated recovery was ranging from 91 to 108%, as shown in Table 4. Hence, Ga_2_O_3_ sensor proved to be valid for determination of Pb^2+^, Cd^2+^ and Hg^2+^ ions in environmental real water samples.

**Table 4:** Analysis of spiked tap water samples for determination of Pb^2+^, Cd^2+^ and Hg^2+^ ions using the Ga_2_O_3_/CPE.

Samples	Heavy metals	Added (µM)	Found (µM)	Recovery %
Tap water 1	Cd^2+^	80	81.44	101
	Pb^2+^	80	72.42	90
Tap water 2	Cd^2+^	80	86.56	108
	Hg^2+^	80	73.84	92
Tap water 3	Pb^2+^	70	69.98	99
Tap water 4	Pb^2+^	70	66.07	94
	Hg^2+^	70	70.12	100

## Conclusion

Ga_2_O_3_ nanoparticles have been chemically synthesized, physically and chemically characterized. Electrochemical characteristics of those nanoparticles showed remarkable conductivity and electro-catalytic activities, thus carbon paste electrodes were modified and suggested for the simultaneous voltammetric determination of multiple heavy metal ions (Pb^2+^, Cd^2+^ and Hg^2+^). A DPV assay was fully optimized; hence, the selectivity, sensitivity, reproducibility, and repeatability were eventually accomplished. Accordingly, direct analysis of environmental samples was conducted without any prior sample preparation, and validated with a reference method (ICP), whereas an excellent recovery was obtained for all tested samples.

## Supplementary Information

Supplementary Information 1.

Supplementary Information 2.

## Data Availability

All data generated or analyzed during this study are included in this published article (and its Supplementary Information files).
