# Supplementary material for: A novel gallium oxide nanoparticles-based sensor for the simultaneous electrochemical detection of Pb2+, Cd2+ and Hg2+ ions in real water samples
Source: Sci Rep. 2022 Nov 23;12:20181. doi: 10.1038/s41598-022-24558-y (PMC9691749; doi:10.1038/s41598-022-24558-y)
Supplement: Supplementary file 1 — Supplementary Information 1. [file 41598_2022_24558_MOESM1_ESM.docx]

**A novel gallium oxide nanoparticles-based sensor for the simultaneous electrochemical determination of Pb^2+^, Cd^2+^ and Hg^2+^ ions in real water samples**

Gehad Abd El-Fatah ^a^, Hend S. Magar ^b^, Rabeay Y. A. Hassan ^c^, Rehab Mahmoud ^a^, Ahmed A. Farghali^d^, Mohamed E.M. Hassouna^a^*

^a^Chemistry Department, Faculty of Science, 62514, Beni-Suef University, Beni-Suef, Egypt

^b^Applied Organic Chemistry Department, National Research Centre (NRC), Dokki, Giza, 12622, Egypt

^c^Nanoscience Program, University of Science and Technology (UST), Zewail City of Science and Technology, Giza 12578, Egypt

^d^Materials Science and Nanotechnology Department, Faculty of Postgraduate Studies

for Advanced Sciences, 62511, Beni-Suef University, Beni-Suef, Egypt

* Corresponding author.

E-mail addresses: Mohamed.hassouna@science.bsu.edu.eg (M.E.M. Hassouna)

**Figure Captions:**

**Figure 1S** DPV plots for the influence of deposition potential in 0.1 M HNO_3_ containing 0.001 M of each of Pb^+2^, Cd^+2^ and Hg^+2^ ions at deposition time 30 s.

**Figure 2S** DPV results for the effect of deposition time in 0.1 M HNO_3_ containing 0.001 M of each of Pb^+2^,Cd^+2^ and Hg^+2^ ions at fixed deposition potential -1.1 V, equilibrium time 15 s and step potential 0.01 V.





**Figure 1S**

**

**

**Figure 2S**
