# Supplementary material for: A novel gallium oxide nanoparticles-based sensor for the simultaneous electrochemical detection of Pb2+, Cd2+ and Hg2+ ions in real water samples
Source: Sci Rep. 2022 Nov 23;12:20181. doi: 10.1038/s41598-022-24558-y (PMC9691749; doi:10.1038/s41598-022-24558-y)
Supplement: Supplementary file 2 — Supplementary Information 2. [file 41598_2022_24558_MOESM2_ESM.docx]

**A novel gallium oxide nanoparticles-based sensor for the simultaneous electrochemical determination of Pb^2+^, Cd^2+^ and Hg^2+^ ions in real water samples**

Gehad Abd El-Fatah ^a^, Hend S. Magar ^b^, Rabeay Y. A. Hassan ^c^, Rehab Mahmoud ^a^, Ahmed A. Farghali^d^, Mohamed E.M. Hassouna^a^*

^a^ Chemistry Department, Faculty of Science, 62514, Beni-Suef University, Beni-Suef, Egypt

^b^ Applied Organic Chemistry Department, National Research Centre (NRC), Dokki, Giza, 12622, Egypt

^c^ Nanoscience Program, University of Science and Technology (UST), Zewail City of Science and Technology, Giza 12578, Egypt

^d^ Materials Science and Nanotechnology Department, Faculty of Postgraduate Studies

for Advanced Sciences, 62511, Beni-Suef University, Beni-Suef, Egypt

*** Corresponding author:**

E-mail addresses Mohamed.hassouna@science.bsu.edu.eg (M.E.M. Hassouna)

| **Electrode type** | ***I_pa_***  **(µ A)** | ***I_pc_***  **(µA)** | **E *_pa_***  **(V)** | **E*_pc_***  **(V)** | **∆E_p_**  **(V)** | **E_1/2_**  **(V)** | **R_s_**  **(Ω)** | **R_ct_**  **(Ω)** | **C**  **(µF)** | **W**  **(Ω)** |
| --- | --- | --- | --- | --- | --- | --- | --- | --- | --- | --- |
| Bare | 136.3 | -125.9 | 0.305 | 0.145 | 0.16 | 0.225 | 53.7 | 207.19 | 6.47 | 1360.4 |
| Ga_2_O_3_ | 230.49 | -246.9 | 0.29 | 0.155 | 0.135 | 0.22 | 42.3 | 98.3 | 9.88 | 955.2 |
| WO_2_ | 212.7 | -164.1 | 0.308 | 0.145 | 0.163 | 0.226 | 33.4 | 159.5 | 9.1 | 1005.6 |
| ZrO_2_ | 195.1 | -198.4 | 0.314 | 0.135 | 0.179 | 0.224 | 32.8 | 183.6 | 7.65 | 1041.9 |
| NiO | 96.2 | -106.4 | 0.301 | 0.151 | 0.150 | 0.226 | 47.2 | 254.1 | 6.15 | 2190.5 |
| CeO_2_ | 69.1 | -69.8 | 0.295 | 0.158 | 0.453 | 0.226 | 42.6 | 382.5 | 3.052 | 3587.3 |

**Table. 1***.* Data analysis of the resulted voltammetric peaks and Nyquist plots of the EIS spectra for electrodes modified with MOs observed in Fig.6 (A) and (B).

I_pa_ : Anodic current (reduction occur) I_pc_ : cathodic current (oxidation occur)

E_pa_: Anodic peak potential E_pc_: cathodic peak potential

E_1/2_ : tha main value of Epa and Epc [=(Epa+Epc)/2]

∆Ep : The difference between the two peak potentials [=Epa-Epc]

Rs : tha resistance of solution Rct : Charge transfer resistance

C: Capacitance W : Warburg resistamce
